# Experimental Study on Dynamic Mechanical Performance of Post-Fire Concrete Confined by CFRP Sheets

**DOI:** 10.3390/ma17092076

**Published:** 2024-04-28

**Authors:** Jingya Liu, Jingsi Huo, Haitao Wang, Zhongping Tang, Qingyan Zhang, Shixiang Yi

**Affiliations:** 1 Institute of Structural Material Failure and Strengthening Technology, Ningbo Polytechnic, Ningbo 315800, China; 2018071@nbpt.edu.cn (J.L.); 2022005@nbpt.edu.cn (Z.T.); 2020007@nbpt.edu.cn (Q.Z.); 13732180686@163.com (S.Y.); 2 College of Civil and Architecture Engineering, Chuzhou University, Chuzhou 239000, China; 3 Fujian Provincial Key Laboratory of Intelligent Infrastructure and Monitoring, Huaqiao University, Xiamen 361021, China; jingsihuo@gmail.com; 4 Engineering Research Center of Low-carbon Highway Building Materials of Anhui Province, Chuzhou 239000, China

**Keywords:** CFRP-confined, post-fire concrete, SHPB, DIF, dynamic mechanical performance

## Abstract

Impact tests on post-fire concrete confined by Carbon Fiber-Reinforced Polymer/Plastic (CFRP) sheets were carried out by using Split Hopkinson Pressure Bar (SHPB) experimental setup in this paper, with emphasis on the effect of exposed temperatures, CFRP layers and impact velocities. Firstly, according to the measured stress-strain curves, the effects of experiment parameters on concrete dynamic mechanical performance such as compressive strength, ultimate strain and energy absorption are discussed in details. Additionally, temperature caused a softening effect on the compressive strength of concrete specimens, while CFRP confinement and strain rate play a hardening effect, which can lead to the increase in dynamic compressive strength by 1.8 to 3.6 times compared to static conditions. However, their hardening mechanisms and action stages are extremely different. Finally, nine widely accepted Dynamic Increase Factor (DIF) models considering strain rate effect were summarized, and a simplified model evaluating dynamic compressive strength of post-fire concrete confined by CFRP sheets was proposed, which can provide evidence for engineering emergency repair after fire accidents.

## 1. Introduction

Fiber-Reinforced Polymer (FRP) is a novel strengthening material composed of continuous polymer fibers impregnated in a synthetic resin matrix. Depending on the use-oriented type of polymer continuous fiber material, FRP can be roughly categorized into three dominating types: Carbon Fiber Reinforced Polymer (CFRP), Glass Fiber Reinforced Polymer (GFRP), and Aramid Fiber Reinforced Polymer (AFRP). Among these FRP materials, CFRP exhibits the highest elastic modulus and tensile strength [1]. FRP is considered as a typical elastic material and offers excellent mechanical performance such as higher specific strength and excellent elastic modulus compared to traditional reinforcement materials like steel plates. In recent years, FRP is utilized on a large scale in the reinforcement of concrete structures, and one commonly engineering application of reinforcement is to externally confine concrete structural components with FRP along the direction of its fibers to enhance compressive strength as well as ductility [1].

Concrete structures damaged by fire can continue to be on active service after reasonable repair and reinforcement. In addition, building structures may experience an impact effect such as explosions, collisions and other dynamic loads during their service life. These complex dynamic loads have the features of instantaneous nature, randomness, and high strain rates compared to typical static loads. Such load conditions pose significant threats to conventional concrete structures and are particularly challenging for fire-damaged concrete structures. However, existing studies on this field have only focused on one separated aspect, in terms of the static mechanical performance of post-fire concrete, the dynamic mechanical performance of concrete, and the static mechanical performance of FRP-confined concrete, and so forth [2].

The mechanical performance of post-fire concrete is influenced by a complex interplay of various influencing factors, but there is a relatively consistent consensus regarding the attenuation law and mechanisms. For ordinary-grade plain concrete, the one of most crucial influencing factors on its residual compressive strength is the temperature of fire. High temperature caused the evaporation of water within the concrete, leading to a looser overall structure and weakened bond between aggregates. As the temperature increased, the residual compressive strength and elastic modulus of concrete generally showed a decreasing trend. Concrete experienced minimal compressive strength loss when subjected to temperatures below 300 °C. Between 200 °C and 300 °C, a phenomenon similar to secondary hydration due to steam curing may occur, leading to a slight increase in the residual compressive strength of concrete. Between 300 °C and 400 °C, there was a decrease in compressive strength by 10% to 20%. Beyond 400 °C, the compressive strength of concrete declined rapidly, with surface cracks appearing. At around 600 °C, surface cracks penetrated through, bond failure of the concrete protective layer occurred, and strength decreased significantly. At temperatures between 700 °C and 900 °C, the compressive strength of concrete was almost entirely lost [3]. Several studies have proposed a post-fire deterioration model for concrete compressive strength [2,3,4,5,6].

**Figure 1:**
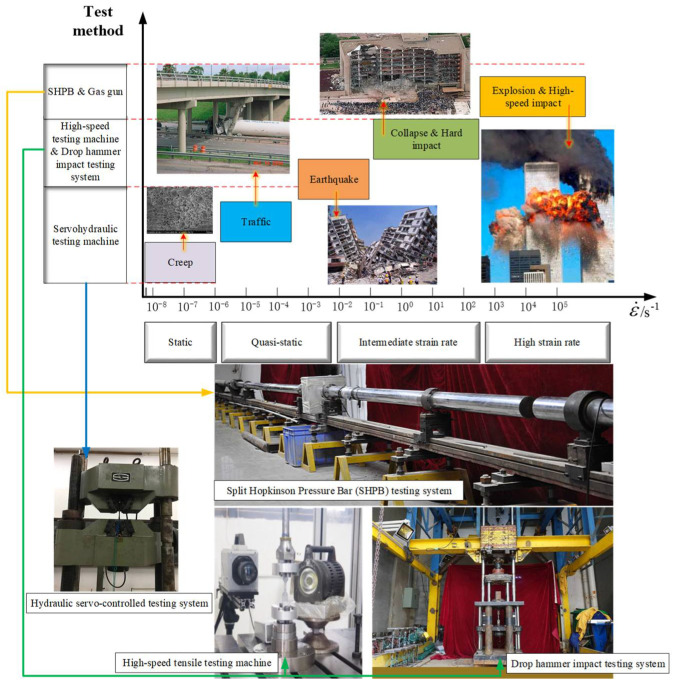
Typical classification of strain rates.

For experimental research on the dynamic mechanical performance of concrete, according to the experimental techniques commonly used to measure the rate-related properties of the material, the strain rate is roughly divided into three regions: low rate, which includes static and quasi-static states (below 10^−2^ s^−1^), intermediate rate (10^−1^ to 10^2^ s^−1^), and high rate (10^3^ to 10^5^ s^−1^). Typically, hydraulic testing machines or similar devices are used to test the mechanical properties of materials at low strain rates, a high-speed testing machine or drop hammer impact testing system are used to test the mechanical properties at an intermediate rate, while SHPB or Gas gun devices are usually used to estimate the mechanical properties at high strain rates, as shown in Figure 1 [7,8]. Due to several prominent advantages in terms of the simplicity of operation, low cost, and excellent testing accuracy, the SHPB test setup has gained widespread application in the field of research on the dynamic mechanical performance of concrete materials [9,10]. In the theoretical study of the dynamic mechanical performance of concrete, one of the hot topics primarily focuses on the Dynamic Increase Factor (DIF), which represents the ratio of dynamic to static compressive strength of concrete. Some scholars propose an exponential relationship between DIF and strain rate, while others suggest a linear or quadratic relationship between the logarithm of DIF and strain rate. There are also theoretical studies stating that the relationship between DIF and strain rate is not fixed, where a critical strain rate at which the form of the DIF model undergoes a change [11,12,13,14,15,16,17,18,19].

Concrete confined by FRP exhibits significant similarities to concrete confined by steel in terms of stress mechanics. However, there are substantial differences in material properties between FRP and steel. FRP displays typical linear elasticity and is considered as a brittle material, whereas steel is an elastic-plastic material. Therefore, substituting FRP for steel in strength directly is not appropriate, and research on the compressive strength model for concrete confined by FRP is indispensable [2]. Teng et al. validated the accuracy of the Lam & Teng model using their own database [1,20,21]. Ozbakkaloglu et al. collected a total of 88 compressive strength models for concrete confined by FRP, comprising more than 2000 test results from about 200 experiments. The mathematical and statistical methods were used to assess the models proposed by previous researchers [22]. The assessment results confirmed the high accuracy of the Lam & Teng model [20,22,23,24,25,26,27,28,29,30].

Few researchers have investigated the dynamic mechanical performance of concrete confined by FRP [31,32,33,34,35,36,37,38,39,40,41]. Zhang et al. conducted dynamic mechanical performance tests on concrete confined by FRP using an electro-hydraulic servo test setup with a strain rate range of 0 to 0.4 s^−1^ [31,32]. Yang et al. used SHPB test setup to investigate the dynamic mechanical performance of concrete confined by FRP with the strain rate range from 50 to 100 s^−1^ [33]. Guedes et al. conducted dynamic mechanical performance tests on concrete confined by FRP at three strain rate levels including 0.0001, 0.07, and 400 s^−1^, using universal testing machine and SHPB test setup [34]. Swesi et al. and Liu et al. used a drop hammer test setup to investigate the performance of concrete columns confined by CFRP under impact loads with strain rates up to 10 s^−1^ [35,36]. These studies have demonstrated that FRP confinement effectively improves the compressive strength and ductility of concrete under dynamic loading conditions [36,37,38,39,40,41]. However, research on the dynamic mechanical performance of post-fire concrete confined by FRP sheets is still lacking in-depth and comprehensive research.

The SHPB test setup was utilized in this paper to investigate the dynamic mechanical performance of post-fire concrete confined by CFRP, with emphasis on the effect of temperatures, CFRP layers and impact velocities on the dynamic mechanical performance of concrete. In addition, this paper proposes a simplified model evaluating the dynamic compressive strength of post-fire concrete confined by CFRP sheets. The research aims to provide experimental evidence for the rational assessment of the mechanical performance of post-fire concrete confined by FRP sheets under complex dynamic loads such as impact and explosion in practical engineering.

## 2. Experimental Program

### 2.1. Test Specimens

The plain concrete specimens were designed for the static test as a comparison group, as shown in Table 1. Besides, in order to minimize the dispersion of waveforms in the SHPB test and achieve a constant strain rate and uniform stress during loading, the optimum length-diameter ratio for SHPB specimens is generally 0.5 [42]. Therefore, a total of 48 concrete specimens with dimensions of Φ94 × 50 mm were designed and fabricated. In order to reduce the dispersion of concrete tests, repeated tests were performed two times per series, as shown in Table 2. In this experiment, the main parameters included the maximum temperature experienced by the core concrete (20 °C, 200 °C, 300 °C, 400 °C, 500 °C, 600 °C), the number of CFRP layers used for external confinement (0 layers, 1 layer, and 2 layers), and the impact velocities (7.5 m/s, and 11.5 m/s), as shown in Table 2. The designed strength grade of concrete was C30, whose mix design was conducted in accordance with Chinese standard [42]. The mechanical performances of the concrete and CFRP were tested according to Chinese standards, respectively [43,44], whose results are shown in Table 3 and Table 4. Carbon fiber reinforcement special adhesive was used between CFRP and concrete.

### 2.2. Experimental Method

Polyvinyl chloride (PVC) round pipes with outer diameter of 110 mm and inner diameter of 94 mm were used as the molds for specimen concreting. The concrete was manually compacted and cured by water sprinkling at room temperature for 28 days. Afterward, the specimens were cut to the desired length, and the outer layer of PVC pipes was removed. The specimens were then left to stand at room temperature for 14 days to allow for sufficient evaporation of surface moisture. In order to achieve the high-temperature test, the specimens were placed in a high-temperature furnace test setup with a temperature controller at Hunan University. For cylindrical ordinary concrete specimens with a height of up to 200 mm and a diameter of up to 100 mm, -there is no temperature gradient on cross-section of concrete after being held at a constant temperature for 3 h [4,45]. Therefore, the temperature was increased automatically at a rate of 10 °C/min until reaching the targeted temperature, which was then maintained for 3 h. After the high-temperature test, the specimens were naturally cooled to room temperature inside the furnace, then taken out and left to stand for 7 days. Following the cooling period, the specimens underwent CFRP wrapping. To prevent premature failure due to insufficient overlap length, the overlap length of CFRP was set to 100 mm according to the Chinese standard [46]. After CFRP wrapping was completed, the specimens were left to stand for an additional 14 days to allow for complete solidification of the bond between CFRP and concrete before specimens subjected to SHPB impact testing.

### 2.3. Test Setup

The experiment was conducted using the self-developed Φ100 mm diameter SHPB test setup at Hunan University, as shown in Figure 1. Different impact velocities were achieved by adjusting the pressure of the impact gas. The impact stress waves were measured using the resistance strain gauges attached to the incident and transmission bars. For a large-diameter SHPB test setup, the transverse contraction and expansion of the bars can cause more significant waveform dispersion [9]. To avoid the waveform dispersion caused by large-diameter bars, a layer of soft cloth was placed at the impacted end of the incident bar to achieve cushion and energy absorbed [9,11,47,48,49].

According to the measured reflected and transmitted waves during the test, stress and strain as well as strain rate of the specimen can be obtained using the two-wave method [11,50,51,52,53]. In addition, the good overlap between typical waveforms of each series of specimens confirms the validity and reliability of the experimental results in the SHPB test, by aligning the incident wave, reflected wave, and transmitted wave at the wavefront [46].

## 3. Experimental Results

### 3.1. Failure Modes

**Figure 2:**
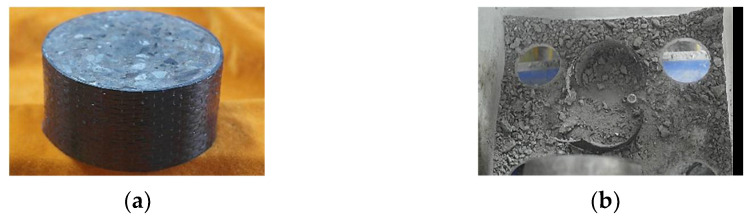
Failure mode of concrete specimen: (**a**) pre-failure specimen; (**b**) post-failure specimen.

During the SHPB impact tests, upon the bullet’s launch, the specimens experienced a rapid and intense impact, resulting in a loud noise. Simultaneously, the concrete has completely shattered, unable to maintain its form, leaving only scattered aggregates. The failure mode observed in all specimens were concrete fragmentation and CFRP fracture, as shown in Figure 2. Under the same tested conditions, the degree of concrete fragmentation increased with higher temperatures. Additionally, more concrete was detached from the fractured CFRP sheets, indicating that the interfacial bond strength between CFRP and concrete exceeded the internal cohesive strength of the concrete due to the high temperature effects. It is noteworthy that CFRP fractures did not occur at the joints, suggesting that the overlap length of CFRP was sufficient, owing to effective CFRP confinement.

The concrete specimens experience an extremely short duration of loading. As the impact stress wave propagates through the concrete, cracks instantaneously appear, penetrating the internal structure of the concrete. There is no gradual crack propagation process. Once CFRP starts to provide confinement, it prevents the lateral deformation of the concrete and maintains its overall integrity. However, when the lateral deformation of the concrete exceeds the ultimate tensile strain of CFRP, CFRP ruptures and loses its confinement effect, which leads to the complete fragmentation of the concrete.

### 3.2. Dynamic Stress-Strain Curves

**Figure 3:**
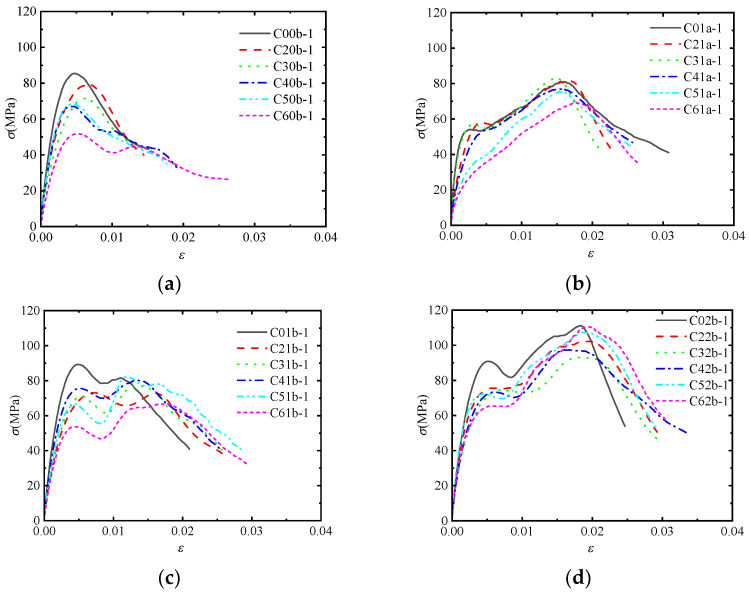
Stress-strain curves of concrete specimens with different temperatures: (**a**) *v* = 11.5 m/s, *n* = 0; (**b**) *v* = 7.5 m/s, *n* = 1; (**c**) *v* = 11.5 m/s, *n* = 1; (**d**) *v* = 11.5 m/s, *n* = 2.

Figure 3 shows the stress-strain curves of the post-fire concrete specimens confined by CFRP sheets with the same number of CFRP layers and impact velocity but at different temperatures. For the post-fire plain concrete, the initial elastic modulus gradually decreases and the shape of the stress-strain curve tends to be flat as the temperature increases. For the post-fire concrete confined by CFRP sheets at low impact velocities, the temperature mainly affects the initial elastic modulus, which shows a decreasing trend. However, this decreasing trend becomes less pronounced at high impact velocities. This phenomenon indicates that the influence of temperature is weakened when the impact load and CFRP confinement act simultaneously.

**Figure 4:**
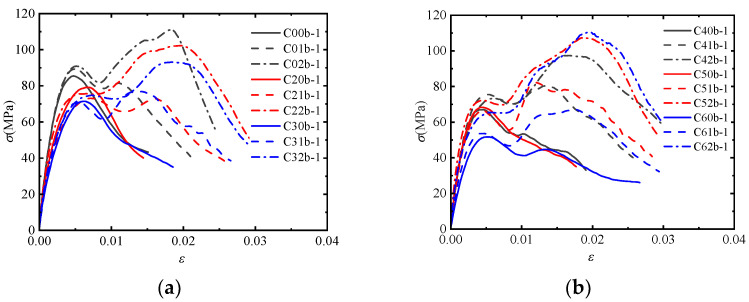
Stress-strain curves of concrete specimens with different CFRP layers (*v* = 11.5 m/s): (**a**) *T* = 20 °C, 200 °C, 300 °C; (**b**) *T* = 400 °C, 500 °C, 600 °C.

Figure 4 shows the stress-strain curves of the post-fire concrete specimens confined by different numbers of CFRP layers at the same temperature and impact velocity. It is observed that the stress-strain curve of the post-fire plain concrete shows a single peak, while the post-fire concrete confined by CFRP sheets shows two peak points. Additionally, there is a softening stage between the two rising segments of the stress-strain curve. This behavior can be attributed to the dynamic failure mechanism of concrete under impact loading. The concrete fractures instantaneously, with cracks appearing directly without a slow development process. At this moment, CFRP and concrete have not yet come into contact, and the confinement effect has no time to occur. Subsequently, the concrete retains its original shape and undergoes lateral expansion under compression. Meanwhile, CFRP starts to provide confinement, leading to a continued increase in the curve. Hence, the first peak point coincides with that of the post-fire plain concrete, which is primarily influenced by the concrete’s own strain rate effect and is independent of the degree of CFRP confinement. CFRP comes into play during the later stage, corresponding to the point that turns from falling to rising in the curve. At this stage, the concrete comes into contact with CFRP, and the lateral deformation of the concrete exceeds which of CFRP. It is evident that the effect of CFRP confinement becomes more pronounced as the temperature increases.

**Figure 5:**
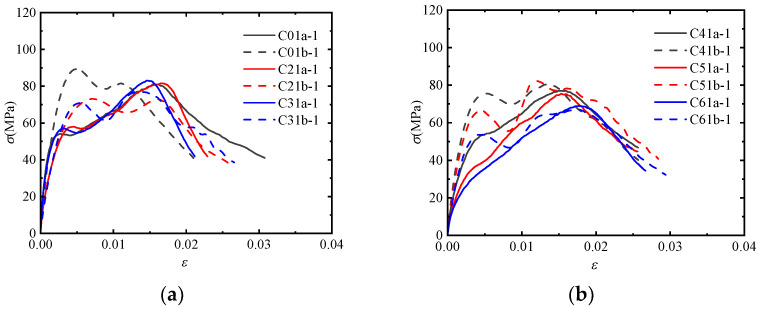
Stress-strain curves of concrete specimens with different impact velocities (*n* = 1): (**a**) *T* = 20 °C, 200 °C, 300 °C; (**b**) *T* = 400 °C, 500 °C, 600 °C.

Figure 5 shows the stress-strain curves of the post-fire concrete specimens under the same temperature confined by one layer of CFRP sheet but under different impact velocities. It can be observed that the concrete’s sensitivity to strain rate leads to a rapid increase in compressive strength and the first peak point during the initial stage of impact, when CFRP has not yet provided confinement. The high impact velocity leads to a higher peak point, while the low impact velocity leads to a lower peak point. Subsequently, the stress-strain curve enters a softening stage, which is less pronounced as the temperature increases at low impact velocities. As the lateral deformation of the concrete develops, CFRP starts to provide confinement, and the stress-strain curve reaches the second peak point, which are coincident with each other under both high and low impact velocities. It is indicated that the first peak point of the stress-strain curve is influenced by the impact velocity. Under the same CFRP confinement, the second peak point of the stress-strain curve remains unchanged. Comparing the two peak points of the stress-strain curve under high impact velocities, it can be seen that the strain rate effect dominates over CFRP confinement effect before 200 °C. However, the dominance is reversed after 200 °C.

## 4. Experimental Parameter Analysis

Table 2 presents SHPB impact test results of post-fire concrete confined by CFRP sheets. How to define dynamic compressive strength, dynamic ultimate strain, and dynamic energy dissipation is a critical issue. Based on the stress-strain curves of the specimens mentioned earlier, it is evident that due to the combined effects of CFRP confinement and strain rate, the stress-strain curves exhibit two stress peak points, namely, *f*_CTD1_ and *f*_CTD2_ in Table 2, corresponding to the strains *ε*_CTD1_ and *ε*_CTD2_, respectively. To analyze these two stress peak points, the average of the two values *f*_CTD, ave_ and *ε*_CTD, ave_ are also provided in Table 2. As for the energy dissipation *S*_CTD_, it is more reasonable to consider the area enclosed by the curve before the second peak point and the coordinate axes. Therefore, the following sections will focus on the analysis of three indicators, including *f*_CTD, ave_, *ε*_CTD, ave_ and *S*_CTD_.

The last three columns in Table 2 also provide the ratios of dynamic to static compressive strength, ultimate strain, and energy dissipation. It is evident that dynamic compressive strength can increase by 1.8 to 3.6 times compared to static conditions, ultimate strain can increase by 1.6 to 7 times, and energy dissipation has increased by 2 to 3 orders of magnitude. This indicates that under the combined effects of CFRP confinement, temperature, and strain rate, the strength and ductility of concrete have significantly improved.

### 4.1. Temperature

**Figure 6:**
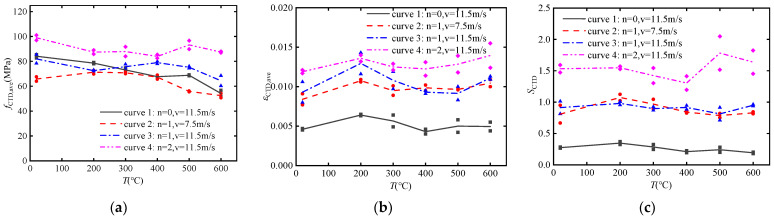
The effect of temperature on dynamic mechanical performance: (**a**) dynamic compressive strength *f*_CTD_; (**b**) dynamic ultimate strain *ε*_CTD_; (**c**) dynamic energy absorption *S*_CTD_.

Figure 6a shows the effect of temperature on the dynamic compressive strength of post-fire concrete specimens. It can be indicated that the softening effect of temperature on the compressive strength of concrete is restricted when subjected to the combined effects of impact load and CFRP confinement. Even for the post-fire plain concrete, the dynamic compressive strength at 600 °C reaches 60% of that at room temperature. With confinement of 2 layers CFRP sheets, the dynamic compressive strength of concrete at 600 °C can even reaches 90% of that at room temperature.

Figure 6b shows the effect of temperature on the dynamic ultimate strain of concrete specimens. It can be observed that the dynamic ultimate strain of post-fire concrete tends to increase with increasing temperature. Post-fire plain concrete under high impact velocity shows relatively stable dynamic ultimate strains, approximately keep in a level of 0.005. However, dynamic ultimate strain of post-fire concrete confined by one layer CFRP sheet under high impact velocity shows a significant upward trend, which is consistent with post-fire concrete confined by CFRP sheets under low impact velocity in the high-temperature range after 400 °C (curve 2 and curve 3). This indicates that temperature has a more pronounced effect on increasing the dynamic ultimate strain of concrete specimens under CFRP confinement and high impact velocity.

Figure 6c shows the effect of temperature on the dynamic energy dissipation capacity of concrete specimens. It can be observed that the energy dissipation capacity of post-fire concrete slightly decreases with increasing temperature (curve 1, 2 and 3), and the difference in impact velocity is not obvious (curve 2 and 3). However, the opposite trend is observed for post-fire concrete confined by two layers of CFRP under high impact velocity, whose energy dissipation capacity slightly increasing with temperature (curve 4). This indicates that the combined effects of CFRP confinement and impact load make the variation of energy dissipation capacity much more complex with different temperature.

### 4.2. CFRP Confinement

**Figure 7:**
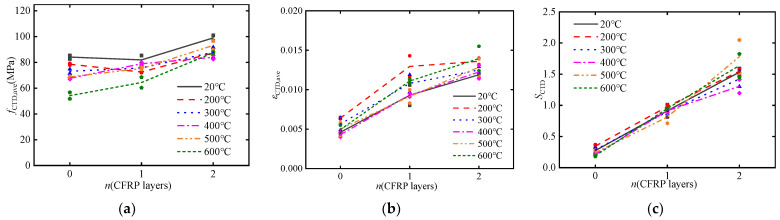
The effect of CFRP confinement on dynamic mechanical performance: (**a**) dynamic compressive strength *f*_CTD_; (**b**) dynamic ultimate strain *ε*_CTD_; (**c**) dynamic energy absorption *S*_CTD_.

Figure 7a shows the effect of CFRP confinement on the dynamic compressive strength of concrete specimens. It can be observed that with an increase in the number of CFRP layers, the dynamic compressive strength of post-fire concrete significantly increases. The increase in dynamic compressive strength is attributed to the contribution of CFRP confinement and the strain rate effect. Furthermore, there is a significant difference in the dynamic compressive strength of post-fire concrete, which decreases noticeably with increasing temperature. However, the difference on dynamic compressive strength of post-fire concrete confined by CFRP sheets is relatively small. This phenomenon also reflects the weakening of the temperature effect due to CFRP confinement.

Figure 7b shows the effect of CFRP confinement on the dynamic ultimate strain of concrete specimens. It can be observed that with an increase in the number of CFRP layers, the dynamic ultimate strain of high-temperature concrete significantly increases. This indicates that CFRP confinement can effectively enhance the ductility of concrete. Furthermore, there is no significant difference in the dynamic ultimate strain of post-fire plain concrete, as it remains relatively stable. However, there is a remarkable difference in the dynamic ultimate strain of post-fire concrete confined by CFRP sheets, especially in the high-temperature range above 400 °C, where it increases noticeably with increasing temperature. This phenomenon suggests that temperature has a certain amplifying effect on the ultimate strain.

Figure 7c shows the effect of CFRP confinement on the dynamic energy dissipation capacity of concrete specimens. It can be observed that CFRP confinement significantly enhances the dynamic energy dissipation capacity of the concrete interface, and show an evident linear relationship.

### 4.3. Strain Rate

Strain rate is an important parameter for measuring impact energy. Research has shown that concrete is a typical strain rate-sensitive material [34]. Currently, there are three main methods for selecting strain rate: (1) peak strain rate, which refers to the strain rate corresponding to the peak stress; (2) average strain rate, which represents the average strain rate during the instantaneous loading process; (3) nominal strain rate, which represents the average strain rate during the entire stress wave propagation process [54]. The peak strain rate is not representative. In addition, the stress wave propagation takes longer compared to the instantaneous impact process, so the nominal strain rate cannot accurately describe the actual strain rate during the loading process. Therefore, this study adopts the average strain rate and approximates the experimental process as constant strain rate loading.

**Figure 8:**
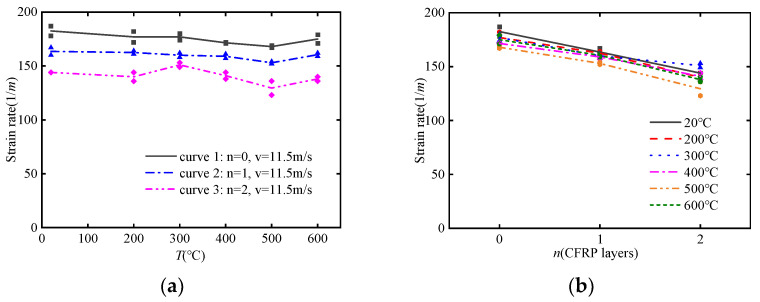
Strain rate curves with different parameters (*v* = 11.5 m/s): (**a**) temperature; (**b**) CFRP layers.

Figure 8 present the variations of average strain rate of CFRP-confined concrete with temperature and the number of CFRP layers, respectively. It can be observed that with an increase in temperature, the average strain rate of concrete slightly decreases, but not significant, as shown in Figure 8a. This indicates that as the temperature rises, the sensitivity of concrete to strain rate gradually decreases. This is mainly attributed to the lateral inertial effect of concrete within the high strain rate range achieved in the SHPB test [11,49,55]. Elevated temperature leads to an increase in microcracks in concrete, resulting in a gradual loosening of the structure and a reduction in internal aggregate cohesion, which weakens the lateral confinement effect and consequently reduces the sensitivity of concrete to strain rate. Additionally, as the number of CFRP layers increases, the strain rate sensitivity of concrete significantly decreases, as shown in Figure 8b. This can be attributed to the strain rate insensitivity of FRP. Previous studies have shown that FRP exhibits negligible sensitivity to strain rate, with its dynamic strength and ultimate strain being comparable to its static properties [56,57]. Compared to concrete, FRP has a low sensitivity to strain rate and can absorb a portion of the energy during the impact process, which can significantly enhance the energy dissipation capacity at the bonded interface. Moreover, the confinement effect of CFRP on concrete effectively restrains its lateral deformation, improves its ductility, and delays its failure time [58].

**Figure 9:**
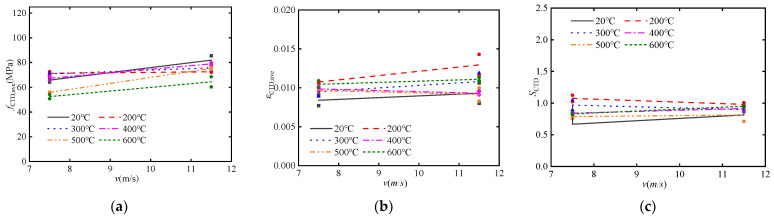
The effect of impact velocity on dynamic mechanical performance: (**a**) dynamic compressive strength *f*_CTD_; (**b**) dynamic ultimate strain *ε*_CTD_; (**c**) dynamic energy absorption *S*_CTD_.

Figure 9a shows the effect of impact velocity on the dynamic compressive strength of concrete specimens. It is evident that dynamic compressive strength significantly increases with the increase in impact velocity. However, this trend is not pronounced at lower temperatures of 200 °C and 300 °C but becomes more apparent at higher temperatures above 300 °C. This is because in the low-temperature range below 300 °C, the first peak point of the stress-strain curve influenced by the strain rate effect is greater than the second peak point influenced by the CFRP confinement effect under high-velocity impact, while the opposite trend is observed under low-velocity impact. Furthermore, it is indicated that the combined effect of the strain rate and CFRP confinement partially mitigates the strain rate effect.

Figure 9b shows the effect of impact velocity on the dynamic ultimate strain of concrete specimens. It can be observed that impact velocity has an increasing effect in the low-temperature range below 300 °C and gradually decreases in the high-temperature range above 300 °C.

Figure 9c shows the impact of impact velocity on the dynamic energy dissipation capacity of concrete specimens. It can be observed that the pattern of energy absorption also follows a division at 300 °C. In the low-temperature range below 300 °C, the energy absorption decreases with increasing impact velocity, while in the high-temperature range above 300 °C, the opposite trend is observed.

## 5. Theoretical Analysis

### 5.1. Summary of DIF Models

Equations (1)–(9) summarize nine widely accepted DIF models in current literature [11,12,13,14,15,16,17,18,19]:

(1) CEB model [13]:

(1) $$DIF=\left\{\begin{array}{c} {\left(\frac{\dot{\varepsilon }}{{\dot{\varepsilon }}_{s}}\right)}^{1.026\alpha }\;\;\;\;\;\;\;\dot{\varepsilon }\leq 30s^{-1}\\ \gamma_{s}{\left(\frac{\dot{\varepsilon }}{{\dot{\varepsilon }}_{s}}\right)}^{1/3}\;\;\;\;\dot{\varepsilon }>30s^{-1} \end{array}\right.$$

In Equation (1), $\alpha ={\left(5+9f_{cs}/f_{co}\right)}^{-1}$; $f_{co}=10\;M$Pa; $\gamma_{s}=10^{6.156\alpha -2}$; $\delta ={\left(10+6f_{cs}/f_{co}\right)}^{-1}$.

(2) Tedesco & Ross model [14]:

(2) $$DIF=\left\{\begin{array}{c} 0.00965log\dot{\varepsilon }+1.058\geq 1.0\;\;\;\;\dot{\varepsilon }\leq 63.1s^{-1}\\ 0.758log\dot{\varepsilon }-0.289\leq 2.5\;\;\;\;\;\;\;\dot{\varepsilon }\geq 63.1s^{-1} \end{array}\right.$$

(3) Grote et al. model [15]:

(3) $$DIF=\left\{\begin{array}{c} 0.0235log\dot{\varepsilon }+1.07\;\;\;\;\;\;\;\;\;\;\;\;\;\;\;\;\;\;\;\;\;\;\;\;\;\;\;\;\;\dot{\varepsilon }\leq 266s^{-1}\\ 0.882{\left(log\dot{\varepsilon }\right)}^{3}-4.4\;{\left(log\dot{\varepsilon }\right)}^{2}\;+7.22log\dot{\varepsilon }-2.64\;\;\;\;\;\;\;\dot{\varepsilon }\geq 266s^{-1} \end{array}\right.$$

(4) Li & Meng model [11]:

(4) $$DIF=\left\{\begin{array}{c} 0.03438\left(3+log\dot{\varepsilon }\right)+1\;\;\;\;\;\;\;\;\;\;\;\;\;\;\;\;\;\;\;\;\;\;\;\dot{\varepsilon }\leq 100s^{-1}\\ 1.729{\left(log\dot{\varepsilon }\right)}^{2}\;-7.1372log\dot{\varepsilon }+8.5303\;\;\;\;\;\;\;\;\;\;\;\;\dot{\varepsilon }\geq 100s^{-1} \end{array}\right.$$

(5) Ngo et al. model [16]:

(5) $$DIF=\left\{\begin{array}{c} {\left(\frac{\dot{\varepsilon }}{{\dot{\varepsilon }}_{s}}\right)}^{1.026\alpha }\;\;\;\;\;\;\;\;\;\;\;\;\dot{\varepsilon }\leq {\dot{\varepsilon }}_{1}\\ A_{1}\ln(\dot{\varepsilon })\;-A_{2}\;\;\;\;\;\;\;\dot{\varepsilon }\geq {\dot{\varepsilon }}_{1} \end{array}\right.$$

In Equation (5), $A_{1}=0.9866-0.0044f_{cs}$; $A_{2}=2.1396-0.0128f_{cs}$; $\alpha =1/\left(20+f_{cs}/2\right)$; ${\dot{\varepsilon }}_{1}=0.0022{f_{cs}}^{2}-0.1989f_{cs}+46.137$.

(6) Katayama et al. model [17]:

(6) $$DIF=0.2583{\left(log\dot{\varepsilon }\right)}^{2}\;-0.05076log\dot{\varepsilon }+1.021$$

(7) Beppu et al. model [18]:

(7) $$DIF={\left(\dot{\varepsilon }/{\dot{\varepsilon }}_{s}\right)}^{0.006{\left[\log\left({\dot{\varepsilon }}_{1}/{\dot{\varepsilon }}_{s}\right)\right]}^{1.05}}$$

In Equation (7), ${\dot{\varepsilon }}_{1}=0.0022{f_{cs}}^{2}-0.1989f_{cs}+46.137$.

(8) Hartmann et al. model [19]:

(8) $$DIF=0.5{\left(\dot{\varepsilon }/{\dot{\varepsilon }}_{0}\right)}^{0.13}+0.90\;\;\;\;\;{\dot{\varepsilon }}_{0}=1s^{-1}$$

(9) Al-Salloum et al. model [12]:

(9) $$DIF=\left(3.54\dot{\varepsilon }+430.6\right)/\left(\dot{\varepsilon }+447.3\right)$$

In Equations (1)–(9), *f*_cs_ represents static compressive strength of concrete; $\dot{\varepsilon }$ represents the strain rate; ${\dot{\varepsilon }}_{s}$ is the quasi-static compressive strain rate. ${\dot{\varepsilon }}_{s}=3\times 10^{-5}s^{-1}$.

**Figure 10:**
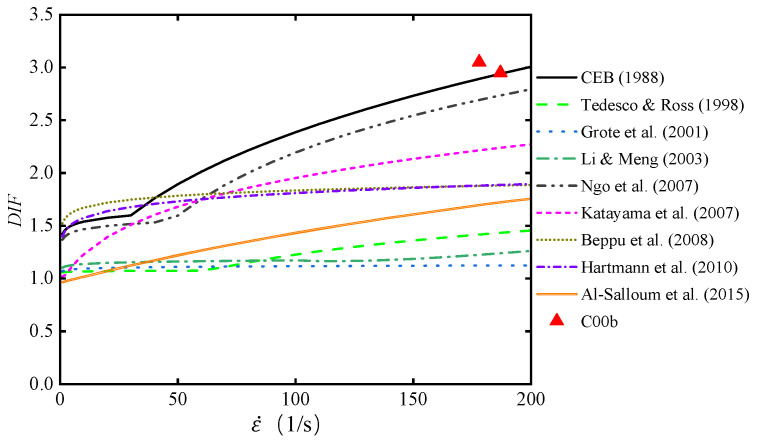
The curves of various DIF models [11,12,13,14,15,16,17,18,19].

Figure 10 depicts the curves of various DIF models. It can be observed that despite all models displaying the same trend of increasing DIF with higher strain rates, the differences between the curves are quite pronounced. This is primarily attributed to variations in experimental conditions, such as testing setups, specimen material properties, and specimen dimensions [50]. Research by Al-Salloum et al. indicated that shorter and more circular specimens yield higher DIF values, while concrete strength has negligible impact on DIF [12]. Lateral confinement is the dominant factor contributing to increased DIF. Strain rate effects become prominent when strain rates are around 100 s^−1^. This apparent strengthening of strain rate effects might be due to the sensitivity of flow pressures induced by the lateral restraint, driven by the material strength [11].

Figure 10 also includes data from the C00b of tests, representing DIF values when strain rate effects act independently. The considerable data scatter makes it challenging to straightforwardly identify the DIF model that best fits the experimental data.

### 5.2. Simplified Model for Dynamic Compressive Strength

In order to propose a simplified model for the dynamic compressive strength of post-fire concrete confined by CFRP sheets, this paper takes into consideration the temperature effect, CFRP confinement effect and concrete strain rate effect, while neglecting the coupling effects among them. It is assumed that the deformation characteristics of concrete remain unchanged throughout the entire process of high temperature and impact tests.

The logical sequence for considering these three effects is crucial. Since concrete is influenced by high temperatures first, the temperature effect should be addressed initially. The CFRP confinement effect and strain rate effect both come into play during the impact process. Based on the analysis of failure mechanisms and parameter analysis discussed earlier, it can be inferred that the concrete strain rate effect begins to manifest in the early loading stages, while CFRP confinement effect operates in the later loading stages. Therefore, the strain rate effect should be considered before addressing CFRP confinement effect.

The compatibility of Li and Guo’s model and Lam and Teng’s model with the test results in this experiment condition has been verified [2,5,20]. In the context of proposing the dynamic compressive strength of post-fire concrete confined by CFRP sheets, their model expressions are still utilized. However, as mentioned earlier, due to limited data, it is not straightforward to determine the DIF model that best fits the experimental data. Hence, various DIF models are used for separate calculations.

According to Li and Guo’s post-fire deterioration model for concrete compressive strength [5], the expression for $f_{TS}$, which represents the static compressive strength of plain post-fire concrete is

(10) $$f_{TS}=\alpha_{t}f_{s}$$

The decay coefficient *α*_t_ of compressive strength for plain post-fire concrete is:

(11) $$\alpha_{t}=\frac{1}{1+{2.4(T-20)}^{6}\times 10^{-17}}$$

In Equations (10) and (11), *T* represents the temperature (°C), and $f_{s}$ denotes compressive strength of concrete in the room temperature, as shown in Table 1.

The expression for $f_{TD}$, which represents the dynamic compressive strength of plain post-fire concrete is:

(12) $$f_{TD}=DIF\cdot f_{TS}=DIF\cdot \alpha_{t}f_{s}$$

Where DIF is calculated using Equations (1)–(9).

As per Lam & Teng model for FRP-confined concrete compressive strength [20], the expression for $f_{CTD}$, which represents the dynamic compressive strength of post-fire concrete confined by CFRP sheets is:

(13) $$f_{CTD}=\alpha_{c}f_{co}^{\prime }=\alpha_{c}f_{TD}=DIF\cdot \alpha_{c}\alpha_{t}f_{s}$$

(14) $$f_{co}^{\prime }=f_{TD}$$

The increase coefficient in compressive strength of concrete confined with CFRP after exposure to high temperatures, denoted as $\alpha_{c}$, is:

(15) $$\alpha_{c}=1+3.3\frac{f_{l}}{f_{co}^{\prime }}$$

(16) $$f_{l}=\frac{2f_{f}t_{f}}{D}$$

In Equations (15) and (16), $f_{co}^{\prime }$ represents the compressive strength of the post-fire concrete core; $f_{l}$ represents the lateral confinement stress of the core concrete; $f_{f}$ and $t_{f}$ represent the tensile strength and nominal thickness of CFRP, respectively, as shown in Table 4; *D* represents the diameter of the core concrete.

Equations (13)–(16) represent the model for the dynamic compressive strength of post-fire concrete confined by CFRP sheets.

**Figure 11:**
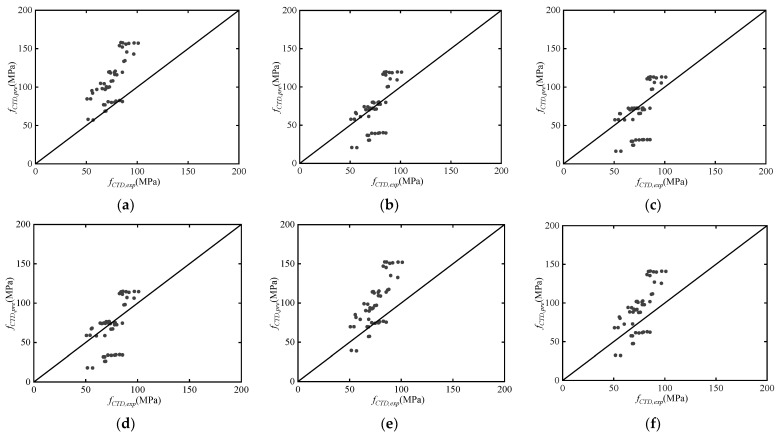

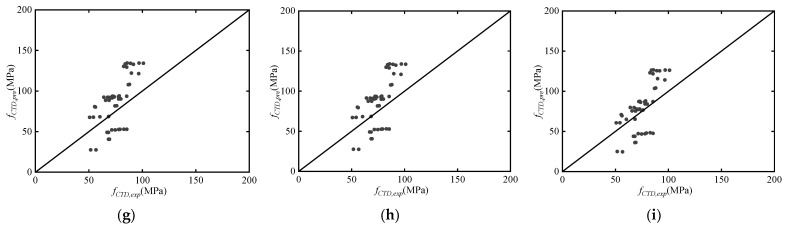
The comparison between calculated values using different DIF models substituted into the simplified model and experimental values: (**a**) CEB model [13]; (**b**) Tedesco & Ross model [14]; (**c**) Grote et al. model [15]; (**d**) Li & Meng model [11]; (**e**) Ngo et al. model [16]; (**f**) Katayama et al. model [17]; (**g**) Beppu et al. model [18]; (**h**) Hartmann et al. model (2010) [19]; (**i**) Al-Salloum et al. model [12].

Figure 11 illustrates the comparison between calculated values using different DIF expressions substituted into the simplified model and experimental values, denoted as $f_{CTD,pre}$ and $f_{CTD,exp}$. Table 5 provides statistical indicators for the ratios between calculated and experimental values. It can be observed that the widely used CEB model demonstrates practicality within the range of medium-to-low strain rates [13]. However, in the experiments conducted in this study, the calculated values using this model significantly exceed the experimental values. This suggests that the CEB model is not suitable for the high strain rate range attainable by SHPB test setup [13]. Through comprehensive comparison of the nine DIF models, it can be noted that Tedesco & Ross model and Al-Salloum et al. model exhibit good agreement with experimental data [12,14]. The average ratio of calculated values to experimental values is closest to 1, and the standard deviation is relatively small, approximately 0.300. Specifically, the Tedesco & Ross model tends to slightly underestimate the experimental values, leaning towards safety, making it suitable for simplified assessments in design [14].

From Figure 11, it can be observed that the proposed simplified model tends to be conservative for 0 and 1 layers of CFRP confinement. The model overestimates the dynamic compressive strength of post-fire concrete confined by CFRP while for 2 layers of CFRP confinement. It becomes evident that CFRP can significantly enhance the dynamic compressive strength as well as ductility of concrete. However, this enhancement is not limitless [59]. In practice, it’s crucial to consider factors such as concrete dimensions and the extent of damage to determine an appropriate number of CFRP layers for external confinement, to avoid unnecessary redundancy in strengthening efforts. Furthermore, the degree of brittle failure of concrete confined by CFRP is significantly increased, and the failure is often sudden without apparent warning, resulting in severe specimen damage [60]. Therefore, in practical engineering applications, direct utilization of the dynamic compressive strength of post-fire concrete confined by CFRP is not straightforward and remains an area that requires further investigation [61]. However, within the parameter range of this experiment, the proposed model exhibits good accuracy and practicality.

## 6. Conclusions

Based on the experimental parameters analysis, the dynamic mechanical performance of post-fire concrete confined by CFRP sheets was investigated in this paper. The following conclusions are drawn:

1. Temperature exposure leads to a negative effect on the compressive strength and initial stiffness of concrete, while it results in the increased ultimate strain and the flat stress-strain curve. In addition, strain rate leads to a positive effect on the compressive strength and initial stiffness of concrete, with a decreasing effect on the ultimate strain at low temperatures and an increasing effect at high temperatures, resulting in a steeper stress-strain curve. Moreover, the number of CFRP layers increases the compressive strength and ultimate strain of concrete, without significantly affecting the initial stiffness, and elongates the stress-strain curve, significantly enhancing the dynamic energy dissipation capacity at the interface.
2. Owing to combined effects of temperature, strain rate, and CFRP confinement, the softening effect of temperature has the least influence, while the strain rate strengthening effect has a secondary influence, and CFRP confinement effect has the tremendous influence. The mechanisms of the latter two are different and affect different points on the stress-strain curve. The strain rate effect primarily acts in the early loading stage, influencing the first peak point of the stress-strain curve. CFRP confinement effect primarily acts in the later loading stage, influencing the second peak point of the stress-strain curve. Both CFRP confinement effect and the temperature effect reduce the strain rate sensitivity of concrete.
3. Based on the existing models, a simplified model for the dynamic compressive strength of post-fire concrete confined by CFRP sheets is proposed by considering the temperature effect together with the concrete strain rate effect and CFRP confinement effect. Through comparison with experimental values, its accuracy and practicality are verified. The research results of this paper can be used as reference for engineering emergency repair after fire accidents.

However, the application scope of CFRP-confined concrete columns has certain limitations. For instance, when the temperature is too high, the concrete has already failed. Therefore, the feasibility and rationality of using CFRP for reinforcement in actual post-fire engineering repairs need to be further evaluated. Additionally, the durability of post-fire concrete confined by CFRP sheets also needs further optimization in construction processes and reinforcement design, such as adding surface coatings and applying prestressing. These aspects should all be reflected in-depth research endeavors in the future.

## Figures and Tables

**Table 1:** Static test results of plain concrete.

Specimen Number	Specimen Size	*T* (°C)	*n* (Layer)	*v* (m/s)	$\dot{\varepsilon }$ (s^−1^)	*f*_S_ (MPa)	*f*_S,ave_ (MPa)	*ε*_S_	*ε*_S,ave_	*S*_S_ (MPa)	*S*_S,ave_ (MPa)
	Φ*D*(mm) × *L*(mm)										
C000−1	Φ94.1×279	20	0	0	6 × 10^−4^	28.34	28.06	0.0020	0.0022	0.030	0.0034
C000−2	Φ94.6×280				6 × 10^−4^	27.77		0.0023		0.038	

Note: 1. In the specimen number, the first letter “C” represents concrete; the first digit “0” represents the temperature as 20 °C; the second digit “0” represents the number of CFRP layers as 0; the third digit “0” represents the impact velocity as 0; the last digit “1”, “2” represents the number of repeated specimens. 2. In the specimen size, *D* and *L* represent the outer diameter and length of the specimen, respectively. 3. In the experimental results, *T* represents the temperature experienced by the specimen; *n* represents the number of external CFRP layers; v represents the impact velocity; $\dot{\varepsilon }$ represents strain rate; *f*_S_ represents static compressive strength, which refers to the highest peak point of the stress-strain curve; *ε*_S_ represents static ultimate strain, which refers to the strain at the compressive strength; *S*_S_ represents the static energy dissipation capacity, which refers to the area enclosed by the stress-strain curve before the highest peak point and the coordinate axis; the subscript “ave” represents the average value.

**Table 2:** SHPB impact test results of post-fire concrete confined by CFRP sheets.

Specimen Number	Specimen Size	*T* (°C)	*n* (Layer)	*v* (m/s)	$\dot{\varepsilon }$ (s^−1^)	*f*_CTD_ (MPa)			*ε*_CTD_			*S*_CTD_ (MPa)	*f*_CTD,ave_/ *f*_S,ave_	*ε*_CTD,ave_/ *ε*_S,ave_	*S*_CTD,ave_/ *S*_S,ave_
	Φ*D*(mm) × *L*(mm)					*f*_CTD1_	*f*_CTD2_	*f*_CTD,ave_	*ε*_CTD1_	*ε*_CTD2_	*ε*_CTD,ave_				
C00b−1	Φ93.2 × 50.4	20	0	11.5	178	85.49	-	85.49	0.0047	-	0.0047	0.283	3.05	2.14	83.24
C00b−2	Φ93.7 × 51.1		0	11.5	187	82.74	-	82.74	0.0045	-	0.0045	0.265	2.95	2.05	77.94
C01a−1	Φ96.1 × 44.3		1	7.5	84	54.04	81.13	67.59	0.0026	0.0155	0.0091	0.950	2.41	4.11	279.41
C01a−2	Φ96.1 × 46.6		1	7.5	86	52.22	75.79	64.01	0.0035	0.0119	0.0077	0.667	2.28	3.50	196.18
C01b−1	Φ96.5 × 51.1		1	11.5	160	89.27	81.55	85.41	0.0049	0.0111	0.0080	0.813	3.04	3.64	89.12
C01b−2	Φ95.8 × 48.8		1	11.5	167	85.79	71.10	78.45	0.0062	0.0149	0.0106	1.013	2.80	4.80	110.29
C02b−1	Φ97.0 × 51.4		2	11.5	144	90.89	111.19	101.04	0.0051	0.0183	0.0117	1.592	3.60	5.32	468.24
C02b−2	Φ96.7 × 50.3		2	11.5	144	88.21	105.62	96.92	0.0064	0.0177	0.0121	1.473	3.45	5.48	433.24
C20b−1	Φ93.5 × 49.5	200	0	11.5	182	79.15	-	79.15	0.0065	-	0.0065	0.369	2.82	2.95	108.53
C20b−2	Φ94.0 × 50.3		0	11.5	172	77.98	-	77.98	0.0063	-	0.0063	0.324	2.78	2.86	95.29
C21a−1	Φ95.7 × 50.3		1	7.5	69	57.98	81.61	69.80	0.0045	0.0167	0.0106	1.023	2.49	4.82	300.88
C21a−2	Φ95.6 × 49.7		1	7.5	68	63.24	82.18	72.71	0.0043	0.0175	0.0109	1.125	2.59	4.95	330.88
C21b−1	Φ96.3 × 50.3		1	11.5	164	73.08	72.90	72.99	0.0069	0.0162	0.0116	1.006	2.60	5.25	107.65
C21b−2	Φ96.2 × 50.2		1	11.5	161	67.70	76.40	72.05	0.0104	0.0181	0.0143	0.954	2.57	6.48	280.59
C22b−1	Φ96.8 × 52.5		2	11.5	136	75.66	102.27	88.97	0.0067	0.0197	0.0132	1.570	3.17	6.00	461.76
C22b−2	Φ96.5 × 50.4		2	11.5	144	66.76	104.92	85.84	0.0068	0.0211	0.0140	1.526	3.06	6.34	448.82
C30b−1	Φ94.2 × 49.7	300	0	11.5	180	71.42	-	71.42	0.0064	-	0.0064	0.324	2.55	2.91	95.29
C30b−2	Φ94.0 × 51.1		0	11.5	174	74.71	-	74.71	0.0049	-	0.0049	0.246	2.66	2.23	72.35
C31a−1	Φ95.7 × 49.4		1	7.5	67	62.16	80.99	71.58	0.0037	0.0164	0.0101	1.045	2.55	4.57	307.35
C31a−2	Φ95.8 × 50.6		1	7.5	67	57.05	83.01	70.03	0.0031	0.0147	0.0089	0.893	2.50	4.05	262.68
C31b−1	Φ96.6 × 51.3		1	11.5	158	70.77	76.85	73.81	0.0052	0.0142	0.0097	0.869	2.63	4.41	255.59
C31b−2	Φ96.0 × 49.4		1	11.5	162	75.29	80.05	77.67	0.0083	0.0155	0.0119	0.925	2.77	5.41	272.06
C32b−1	Φ97.2 × 49.4		2	11.5	153	74.81	93.17	83.99	0.0073	0.0184	0.0129	1.302	2.99	5.84	382.94
C32b−2	Φ97.8 × 48.8		2	11.5	149	80.80	102.64	91.72	0.0052	0.0190	0.0121	1.544	3.27	5.50	454.12
C40b−1	Φ93.7 × 51.5	400	0	11.5	172	67.01	-	67.01	0.0046	-	0.0046	0.226	2.39	2.09	66.47
C40b−2	Φ93.7 × 51.7		0	11.5	171	68.33	-	68.33	0.0040	-	0.0040	0.196	2.44	1.82	57.65
C41a−1	Φ95.8 × 49.5		1	7.5	69	54.27	77.01	65.64	0.0053	0.0151	0.0102	0.856	2.34	4.64	251.76
C41a−2	Φ95.6 × 49.3		1	7.5	67	57.91	79.94	68.93	0.0053	0.0137	0.0095	0.826	2.46	4.32	242.94
C41b−1	Φ96.1 × 50.7		1	11.5	161	75.57	80.31	77.94	0.0049	0.0133	0.0091	0.885	2.78	4.14	260.29
C41b−2	Φ95.7 × 50.3		1	11.5	157	78.06	81.84	79.95	0.0049	0.0141	0.0095	0.945	2.85	4.32	277.94
C42b−1	Φ98.1 × 50.5		2	11.5	138	73.38	97.39	85.39	0.0061	0.0167	0.0114	1.196	3.04	5.18	351.76
C42b−2	Φ97.2 × 50.1		2	11.5	144	60.54	104.56	82.55	0.0063	0.0198	0.0131	1.414	2.94	5.93	415.88
C50b−1	Φ93.4 × 52.4	500	0	11.5	167	68.35	-	68.35	0.0042	-	0.0042	0.202	2.44	1.91	59.41
C50b−2	Φ93.6 × 50.2		0	11.5	169	69.24	-	69.24	0.0058	-	0.0058	0.280	2.47	2.64	82.35
C51a−1	Φ96.3 × 51.4		1	7.5	69	36.87	75.49	56.18	0.0038	0.0153	0.0096	0.753	2.00	4.34	221.47
C51a−2	Φ95.8 × 49.9		1	7.5	82	41.31	69.64	55.48	0.0032	0.0161	0.0097	0.820	1.98	4.39	241.18
C51b−1	Φ95.9 × 51.8		1	11.5	152	66.67	82.14	74.41	0.0044	0.0122	0.0083	0.712	2.65	3.77	209.41
C51b−2	Φ95.5 × 51.8		1	11.5	154	69.39	82.67	76.03	0.0055	0.0144	0.0100	0.914	2.71	4.52	268.82
C52b−1	Φ96.8 × 51.8		2	11.5	136	72.24	107.34	89.79	0.0046	0.0190	0.0118	1.516	3.20	5.36	445.88
C52b−2	Φ97.5 × 49.9		2	11.5	123	74.15	119.12	96.64	0.0045	0.0232	0.0139	2.048	3.44	6.30	602.35
C60b−1	Φ93.7 × 49.4	600	0	11.5	179	51.70	-	51.70	0.0055	-	0.0055	0.203	1.84	2.50	59.71
C60b−2	Φ93.5 × 51.3		0	11.5	171	56.69	-	56.69	0.0044	-	0.0044	0.182	2.02	2.00	53.53
C61a−1	Φ96.4 × 51.5		1	7.5	76	32.51	68.95	50.73	0.0040	0.0177	0.0109	0.814	1.81	4.93	239.41
C61a−2	Φ95.9 × 51.4		1	7.5	75	35.76	72.71	54.24	0.0032	0.0167	0.0100	0.845	1.93	4.52	248.53
C61b−1	Φ96.8 × 51.1		1	11.5	162	53.62	66.98	60.30	0.0047	0.0178	0.0113	0.937	2.15	5.11	275.59
C61b−2	Φ96.0 × 50.3		1	11.5	159	60.04	76.89	68.47	0.0054	0.0164	0.0109	0.963	2.44	4.95	283.24
C62b−1	Φ97.7 × 48.6		2	11.5	140	65.47	110.48	87.98	0.0054	0.0193	0.0124	1.452	3.14	5.61	427.06
C62b−2	Φ98.1 × 50.3		2	11.5	136	62.17	111.60	86.89	0.0069	0.0241	0.0155	1.824	3.10	7.05	536.47

Note: 1. In the specimen number, the first letter “C” represents concrete; the first digit “0”, “2”, “3”, “4”, “5”, “6” represents the temperature as 20 °C, 200 °C, 300 °C, 400 °C, 500 °C, 600 °C; the second digit “0”, “1”, “2” represents the number of CFRP layers as 0 layers, 1 layer, 2 layers; the second letter “a”, “b” represents the impact velocity as 7.5 m/s and 11.5 m/s; the last digit “1”, “2” represents the number of repeated specimens. 2. Specimen size refers to the size of concrete specimens confined by CFRP sheets, which means the sum of original size and the thickness of CFRP sheets. 3. In the experimental results, *f*_CTD_ and *ε*_CTD_ represent the dynamic compressive strength and the dynamic ultimate strain of specimen, respectively; *S*_CTD_ represents the dynamic energy dissipation capacity, which refers to the area enclosed by the stress-strain curve before the second highest peak point and the coordinate axis; the subscript “1”, “2” represent two peak points of the stress-strain curve; the subscript “ave” represent the average value of two peak points.

**Table 3:** Mix proportion and mechanical properties of concrete.

Strength Grade	Mix Proportion (kg/m^3^)				*f*_cu_ (MPa)		*f*_c_ (MPa)		*E*_c_ (MPa)
	Water	Cement	Sand	Stone	Average Value	Standard Deviation	Average Value	Standard Deviation	
C30	204	402	664	1130	38.4	0.57	29.6	0.98	3.36 × 10^4^

Note: *f_cu_* and *f_c_* represent cube crushing strength and cylinder crushing strength, respectively; *E_c_* represents elasticity modulus.

**Table 4:** Mechanical properties of CFRP.

Model	Fiber Weight (g/m^2^)	*t*_f_ (mm)	*f*_f_ (MPa)		*E*_f_ (MPa)	*δ*_f_(%)
			Average Value	Standard Deviation		
HITEX−C300	300	0.167	3587	5.72	2.36 × 10^5^	1.52

Note: *t*_f_ represents nominal thickness; *f*_f_ represents tensile strength; *E*_f_ represents elasticity modulus; *δ*_f_ represents elongation at break.

**Table 5:** Statistical indicators for the ratios between calculated and experimental values.

DIF Models	CEB [13]	Tedesco & Ross [14]	Grote et al. [15]	Li & Meng [11]	Ngo et al. [16]	Katayama et al. [17]	Beppu et al. [18]	Hartmann et al. [19]	Al-Salloum et al. [12]
Average Value	1.444	0.959	0.897	0.927	1.319	1.208	1.139	1.134	1.039
Standard Deviation	0.261	0.306	0.325	0.323	0.282	0.298	0.320	0.317	0.299

## Data Availability

All the data are available within the manuscript.
